# Quantitative susceptibility mapping of deep brain nuclei in 22q11.2 deletion syndrome

**DOI:** 10.3389/fpsyt.2025.1652700

**Published:** 2026-01-09

**Authors:** Nestor Muñoz, Marisleydis García, Analía Cuiza, Angeles Tepper, Javiera Vásquez, Juan Pablo Ramirez-Mahaluf, Daniella Barbagelata, Juan Aguirre, María Elisa Maldonado, Claudia Ornstein, Rosemarie Fritsch, Gabriela Repetto, Carlos Milovic, Marcelo E. Andia, Nicolas A. Crossley, Cristian Tejos

**Affiliations:** 1 Biomedical Imaging Center, Pontificia Universidad de Chile, Santiago, Chile; 2 Department of Electrical Engineering, Pontificia Universidad de Chile, Santiago, Chile; 3 Millennium Institute for Intelligent Healthcare Engineering, Santiago, Chile; 4 Biomedical Engineering, Faculty of Engineering, Universidad de Santiago de Chile, Santiago, Chile; 5 Department of Psychiatry, Pontificia Universidad Catolica de Chile, Santiago, Chile; 6 Department of Psychiatry and Mental Health, Clinical Hospital, Universidad de Chile, Santiago, Chile; 7 Center for Genetics and Genomics, Facultad de Medicina, Clinica Alemana Universidad del Desarrollo, Santiago, Chile; 8 Radiology Department, School of Medicine, Pontificia Universidad Católica de Chile, Santiago, Chile

**Keywords:** 22q11.2 deletion syndrome, magnetic susceptibility, MRI, iron, deep-brain nuclei

## Abstract

**Background:**

22q11.2 Deletion Syndrome (22q11.2 DS) confers a high risk to dopamine-related disorders such as schizophrenia and Parkinson’s disease. These disorders have recently been associated with abnormal iron concentrations in deep brain nuclei. In this study we hypothesized that abnormal iron concentrations may also appear in deep brain nuclei of individuals with 22q11.2 DS.

**Methods:**

We analyzed iron concentrations in four dopamine-related nuclei (caudate, putamen, substantia nigra, and globus pallidus) of 32 individuals, including adolescents and adults, carriers of the 22q11.2 DS and 49 healthy controls. For all individuals, we characterized iron concentrations in each region by quantifying R2* values and using a recently developed technique called Quantitative Susceptibility Mapping (QSM). We used linear mixed models to analyze potential differences between 22q11.2 DS individuals and our control group, considering brain region, age, sex, laterality, volume size, and framewise-displacement as fixed-effect covariates and individuals’ intercepts as random effects.

**Results:**

All individuals showed age-related increases in R2* values and susceptibility within dopaminergic nuclei (caudate, putamen, and substantia nigra). However, individuals with 22q11.2 DS showed a significantly lower rate of increase compared to healthy control group. This suggests that, over time, individuals with 22q11.2 deletion syndrome accumulate less iron in these nuclei than healthy controls.

**Conclusions:**

Individuals with 22q11.2 DS present lower iron accumulation in dopaminergic areas, such as substantia nigra, caudate and putamen, relative to healthy controls. These findings suggest a possible association between a dopaminergic dysfunction and abnormal iron accumulation.

## 1 Introduction

22q11.2 deletion syndrome (22q11.2 DS) is a neurogenetic disorder caused by a microdeletion of approximately 1.5 to 3 megabases on the long arm of chromosome 22 (1, 2). It is the most frequent microdeletion in humans, with an estimated incidence of 1 in every 6000 births (3). This condition results in a heterogeneous clinical presentation, increasing the risk of cardiac abnormalities, immune and autoimmune diseases, genitourinary and gastrointestinal problems (2). Attention deficit has been also identified in individuals with 22q11.2DS, which was linked to poor social skills in children with the deletion (1). Anxiety symptoms are also observed in early childhood, often escalating during adolescence (1). A decline in intellectual quotient (IQ) over time has been noted, affecting both social norms and cognitive abilities (1). There is also an increased awareness that neuropsychiatric presentations are prominent in adulthood. This microdeletion confers the highest known genetic risk to schizophrenia (2, 4), and is also associated with higher rates of early-onset Parkinson’s disease (5).

The higher risk of individuals with 22q11.2 DS to both psychotic disorders and Parkinson’s disease, suggests the presence of a deep brain nuclei dysfunction, possibly involving dopamine pathways. A recent structural study based on Magnetic Resonance Imaging (MRI) showed changes in the volume and shape of subcortical structures in 22q11.2 DS individuals (6). Structural connections from deep brain regions involved in dopaminergic transmission to the cortex have been also found to be abnormal (7). These changes also appear to have functional consequences, with reported differences in functional connectivity of striatal regions (8, 9). Imaging studies have also explored the neurochemical composition of deep brain nuclei in these individuals. Even though 22q11.2 DS participants have shown normal glutamatergic levels in striatal regions (10), they have marked differences in striatal uptake of ^11^C-DTBZ, a radioligand that binds to VMAT2. This protein is responsible for the transport of cytosolic dopamine (11). The 22q11.2 DS has also been associated with increased pre-synaptic dopamine with PET, with a decrease being found in individuals with the 22q11.2 duplication (12).

Recently, neuropsychiatric disorders linked to dopaminergic dysfunction—such as Parkinson’s disease and schizophrenia—have been examined from the complementary perspective of iron homeostasis abnormalities in deep brain nuclei. Iron is essential for dopamine synthesis, acting as a cofactor for tyrosine hydroxylase, the rate-limiting enzyme in dopamine production (13, 14). However, excessive or mislocalized iron can catalyze redox reactions that promote oxidative stress and dopaminergic neuron vulnerability (15). The interaction between iron and dopamine is now understood as bidirectional and tightly regulated rather than purely toxic: dopamine metabolism generates reactive species capable of reducing Fe³^+^ to Fe²^+^, while iron modulates dopamine oxidation kinetics and contributes to the formation of neuromelanin (13, 16, 17). Neuromelanin itself acts as both a chelator and a buffer, sequestering excess iron and mitigating oxidative stress under physiological conditions, but potentially releasing iron under pathological states such as aging or neurodegeneration (14, 18). Therefore, the iron–dopamine axis represents a dynamic equilibrium involving synthesis, storage, and detoxification pathways, rather than a simple oxidative reaction leading directly to cell death.

Consistent with this view, alterations in iron–dopamine regulation have been implicated in diseases with opposite dopaminergic phenotypes. In Parkinson’s disease, several QSM and post-mortem studies have shown increased iron accumulation in the substantia nigra (SN) and striatum, possibly contributing to dopaminergic neuronal loss (19, 20). In contrast, recent multimodal imaging studies combining QSM and PET have reported reduced iron content and striatal hyperdopaminergia in schizophrenia (21, 22). These findings suggest that both hypo- and hyperdopaminergic states may emerge from dysregulation of the iron–dopamine homeostasis across disease trajectories.

Given the reported associationand between 22q11.2 DS and neuropsychiatric disorders such as psychosis and Parkinson’s disease and considering that these two disorders have been linked to alterations in brain iron levels, it may be hypothesized that 22q11.2 DS is also associated with abnormal patterns of brain iron accumulation or distribution. Although there are some studies reporting systemic iron abnormalities (23, 24), we have not found any previous work evaluating brain iron concentrations in individuals with 22q11.2 DS. In this study, we aimed to identify potential changes in iron concentrations in dopamine-related brain regions (particularly in iron-rich ones) in individuals with 22q11.2 DS using Quantitative Susceptibility Mapping (QSM) MRI. This technique allows quantifying the magnetic susceptibility of brain tissues from the phase of a Gradient Recalled Echo (GRE) MRI image (25, 26). Magnetic susceptibility is a physical property that quantifies the magnetization response of matter to an applied external magnetic field. QSM has been used to detect changes produced by diamagnetic sources like myelin and calcium (which exhibit negative susceptibility), and paramagnetic sources such as iron (which exhibit positive susceptibility) (27–30).

We hypothesized that individuals with 22q11.2 DS would show magnetic susceptibility changes (and therefore changes in iron concentrations) in dopaminergic nuclei (i.e., caudate, putamen), and particularly those rich in iron, such as the SN. Additionally, we analyzed the globus pallidus as a control region of magnetic susceptibility changes.

## 2 Methods

### 2.1 Study populations

We invited to participate individuals with the 22q11.2 DS, aged between 15 and 60 years old, who had been included in previous studies of our group (31) or were contacted via support groups (“Fundación Chilena del Niño con Síndrome Velocardiofacial”). Diagnosis of 22q11.2 DS was confirmed in all patients using multiplex ligation-dependent probe amplification (MPLA). Individuals who had a contraindication for an MRI were excluded. Healthy controls aged 15 to 32 years old, who had no current psychiatric disorder and no lifetime history of a psychotic disorder or other neurological disorder, were also included. Absence of 22q11.2 DS was also confirmed with MLPA in all controls. All participants were assessed using the Mini-international neuropsychiatric interview (MINI) (32). IQ was assessed using the Wechsler Adult Intelligence Scale – Fourth Edition (WAIS-IV) (33). All individuals had the capacity to consent or assent (in case of minors) to participate in the study. The study was approved by the Ethics committee of the Pontificia Universidad Catolica de Chile.

### 2.2 MRI acquisitions

MRI studies were performed in a 3T Philips Ingenia scanner at the Pontificia Universidad Catolica de Chile (Santiago, Chile). Susceptibility images were obtained from a T2*-weighted Gradient recalled multi-echo (T2*-w GRE) sequence with 5 echoes equally spaced, with the following acquisition parameters: repetition time (TR)/echo time (TE) 43.1ms/7.2ms; inter-echo spacing 6.2ms; voxel size 0.59×0.59×1 mm3; matrix size 352×352×160; flip angle of 17°. Two additional sequences were acquired: a structural T1-weighted TFE echo (TR/TE 7.78ms/3.55ms; voxel size 0.5×0.5×1 mm3; matrix size 480×480×341; and a flip angle of 8°), and a functional MRI (fMRI) (total scan time 8.33 min, single shot echo-planar imaging (EPI), TR/TE 2.5s/35ms, flip angle of 82°, field of view 220×220×110 mm and isotropic voxel size of 2.75mm3). T1-weighted and fMRI images were used respectively for normalization to a standard atlas template and to assess in-scanner patient movement as described below.

### 2.3 Image processing and analysis

To obtain the QSM maps we performed the following sequential process, similar as that used in (34). We first removed the skull from the magnitude images using FSL’s Brain Extraction Toolbox (BET), obtaining only the brain tissue on the MRI images (35). We unwrapped the phase signal of each echo using a Laplacian algorithm (36). We removed the background field contributions using three consecutive procedures: applying the Laplacian Boundary Value (LBV) algorithm (37), performing a polynomial fit subtraction to remove transmit/receiver offsets, and applying the variable Sophisticated Harmonic Artifact Reduction for Phase data (vSHARP) algorithm (38). After obtaining the off-resonance field from each of the five echoes, we combined them by a weighted average, considering the T2* magnitude as the weight. The final QSM map was obtained after a dipole inversion using the FANSI toolbox developed by our group (39), with a regularization parameter found by an L-curve approach (40). FANSI toolbox is one of the algorithms recommended by the consensus of the ISMRM electro-magnetic tissue properties study group (26), It generates similar QSM maps to standard methods such as Morphology Enabled Dipole Inversion (MEDI) (41) but faster, reducing computation time.

T2*-w GRE images were co-registered to the structural T1 and normalized to MNI space using a non-linear transform available in the Statistical Parameter Mapping (SPM) toolbox running MATLAB (42). Regions of interest (ROIs) were defined using the Multi-contrast PD25 atlas (43–45). For the purposes of our study, we focused our analysis on the regions involved in the nigrostriatal dopaminergic pathway (46) including the bilateral SN and the striatum divided into caudate and putamen. Additionally, we studied the globus pallidus considering our previous results from a group of individuals with first-episode psychosis (47). Finally, we calculated the volume of each bilateral region with all individuals in the original space (i.e., not in the MNI space).

Since QSM is sensitive to changes in both diamagnetic and paramagnetic sources (in parts per million, ppm), a higher susceptibility may be the result of a reduction of diamagnetic sources or an increase in paramagnetic sources. Similarly, a decrease in susceptibility may be the result of an increase of diamagnetic sources or a reduction of paramagnetic sources. To disentangle these uncertainties, QSM values are commonly analyzed in conjunction with R2* values. This way, a higher iron concentration can be observed as an increase in the QSM and R2* values, whereas a lower iron concentration can be observed as a decrease in the QSM and R2* values. A reduced susceptibility and an increased R2* values reflect an increase in diamagnetic concentrations (e.g., calcium). Finally, an increased susceptibility and a reduced R2* values reflect a decrease in diamagnetic concentrations (e.g., myelin) (29, 48).

We therefore characterized any QSM difference with its corresponding R2* value. These R2* maps were obtained from the magnitude signal of the T2*-w GRE images. R2* maps were computed using a non-linear approach that included a total variation-based regularization. This software is also available in the FANSI toolbox (39).

Data were visually inspected to detect motion artifacts and individuals with gross movement were excluded from the study. We also measured motion during the scanning session by calculating the framewise-displacement (FD) from fMRI acquisitions (49), which was included as a covariate in our general linear model, as described below.

### 2.4 Statistical analysis

Linear mixed effects (LME) models are statistical models that incorporate both fixed effects (often referred to as marginal effects) and random effects (50, 51). These models are used to describe the relationship between a parameter and covariates in grouped data according to one or more classification factors, e.g., longitudinal data, repeated measures data and multilevel data (52). Fixed effects in these models provide information about the overall impact of a parameter on the entire population. In other words, the fixed effects estimate the average effect of a variable, assuming it is consistent across all individuals (53). On the other hand, random effects analyze the variations for each individual or unit, acknowledging that these effects can differ across the population (51, 53). By considering both fixed and random effects, LMEs allow us to determine significant changes between groups and whether specific variables have a significant impact on an outcome of interest. To assess the LME model (50), we divided the statistical analysis into three consecutive models as used by Garcia et al. (47):

Model 1: We defined a general LME model (Equation 1) with several independent variables (i.e. fixed effects) and their interactions. This included factors such as age, brain region, group, sex, FD, region volume, laterality (left or right brain hemisphere), and their interactions. In addition, we considered an intercept for each subject (1 | subject) as a random effect of our LME model (52).

(1) QSM~age+ region+group+sex+FD+volume+laterality+interactions+(1|subject)

Due to the complexity of this model, we first performed a backward elimination to identify the significant predictor variables of the magnetic susceptibility. The backward elimination was based on a marginal Wald test (52).

Model 2: After eliminating non-significant variables of the model, we evaluated the robustness of the obtained inference model (shown in Equation 2) with the implied correlation assumption under the random intercept model (54) providing the coefficients of a semiparametric marginal model fitted via Generalized Estimating Equations (GEE) (55). Through this model, we analyzed the variations of each one of the resulting variables, i.e., if there exists a significant difference in the magnetic susceptibility analyzed by individual variables (e.g., region), having the SN as a reference. Finally, we confirmed the robustness of our model (i.e., the significance of the obtained QSM differences and the associated significant variables), when the obtained marginal model and the random effect model showed similar results.

(2) QSM~significant variables+significant interactions+(1|subject)

Model 3: Finally, we analyzed our resulting linear mixed model for QSM stratified by region as in Equation 3.

(3) $$\begin{array}{c} QSM_{region}\sim \;significant\;variables+significant\;interactions+(1\text{|subject}) \end{array}$$

We repeated the same three-model strategy to analyze R2* values.

To have a global perspective of the behavior of QSM and R2* we also performed a linear regression with the age and each group (control group or 22q11.2 DS). Additionally, we looked for a possible difference between the volume of each region, using a Mann Whitney U test. Finally, to corroborate our findings, in each group we performed an ANCOVA test, and with a linear regression between QSM and R2* values, and the age, group, sex and volume as independent variables.

All the statistical analyzes were performed using the “nlme” (52) and “geepack” (56) packages in R version 4.2.0. A p-value < 0.01 was considered statistically significant as is commonly used in statistical tests (57). To account for multiple comparisons, we corrected p-values using the False Discovery Rate (FDR) (58) approach, also with a p-value of 0.01.

## 3 Results

**Figure 1:**
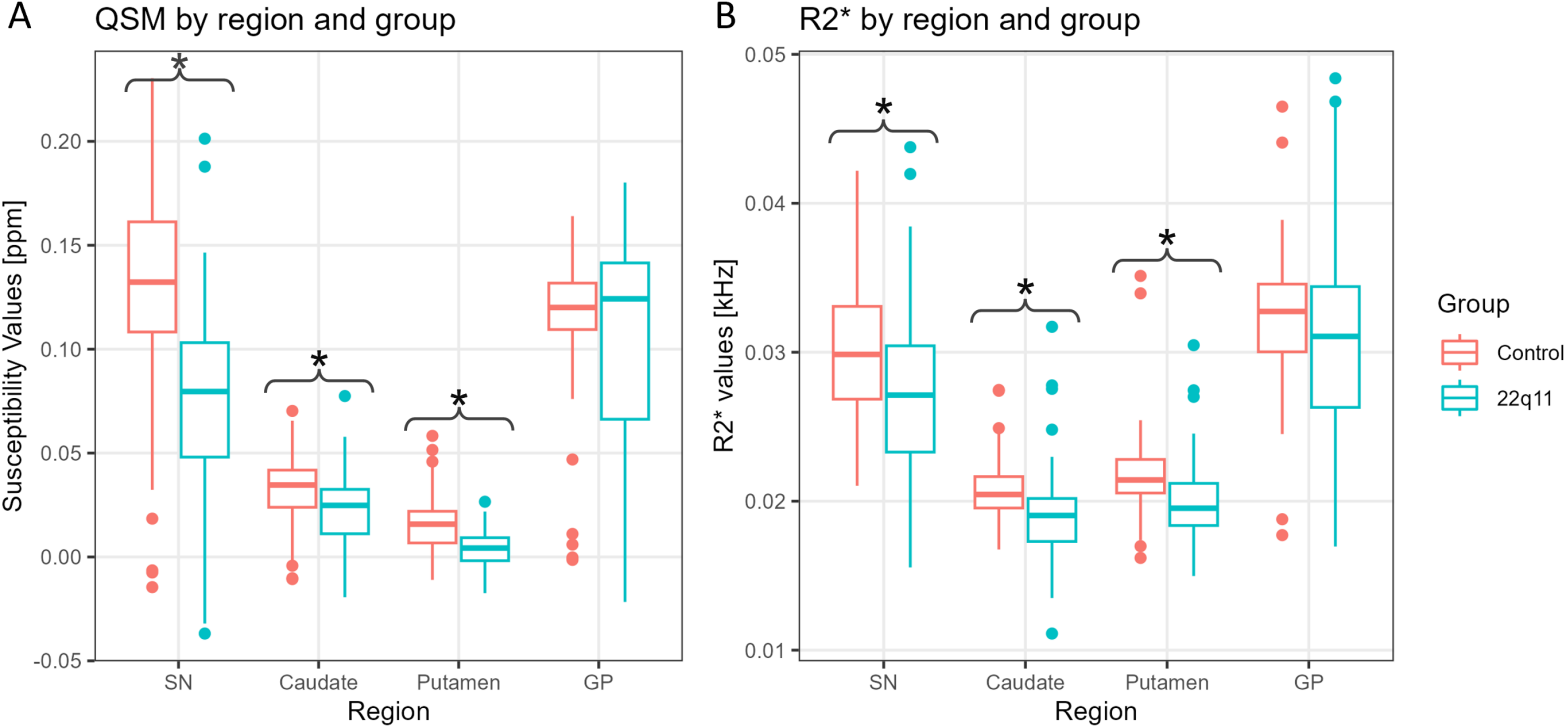
Boxplots of the susceptibility values **(A)** and R2* values **(B)** separated by region and group: healthy controls (red) and 22q11.2 DS individuals (blue). Significant differences are marked with an (*).

Eighty-nine participants were recruited in our study, including 38 individuals with 22q11.2 DS and 51 healthy controls. Six individuals with 22q11.2 DS and two healthy controls were excluded due to excessive head motion during the QSM acquisition, leading to a final cohort of 32 individuals with 22q11.2 DS and 49 healthy controls. **Table 1** shows the demographic data of the participants included in the analysis. Six individuals with 22q11.2 DS had experienced a psychotic episode in the past, were currently in remission according to established clinical criteria (59) and receiving treatment with antipsychotics. Another individual with 22q11.2 DS was acutely psychotic when assessed and was antipsychotic naïve. Boxplots of the susceptibility values for each brain region in healthy controls and 22q11.2 DS individuals appear in **Figure 1**.

**Table 1:** Demographic data of the studied individuals. Number of individuals, their age and number of women with the corresponding study group.

Variable	Healthy subject	22q11.2 DS individuals	Analyses (p values)^a^
Number ^b^	49 (60.5%)	32 (39.5%)	–
Age (years) ^c^	23.61 ± 3.87	23.03 ± 8.02	0.91
Women	15 (45.5%)	18 (54.5%)	0.17
Deletion size	–	AB = 15.4% AD = 69.2% No information = 15.4%	–
Intellectual coefficient ^c^	108.25 ± 12.85	64.05 ± 10.89	–

a *P-values* for *t*-tests for continues variables and Chi-Square for dichotomous variables.

b Number of individuals in each group (percentage of the total number of individuals).

c Mean ± SD of age and intellectual coefficient.

Model 1: The backward analysis revealed that, for susceptibility, region and age were significant predictors, together with the interactions region-age, group-age and region-group-age (**Table 2**), leading to the model.

**Table 2:** Significant predictor variables for magnetic susceptibility and R2* obtained from the backward analysis.

Factor	F-value	P-value
QSM		
(Intercept)	4.73	0.03
Region	42.67	**<0.01**
Age	60.36	**<0.01**
Region*Age	24.53	**<0.01**
Group*Age	70.18	**<0.01**
Region*Group*Age	27.06	**<0.01**
R2*		
(Intercept)	205.62	**<0.01**
Region	22.72	**<0.01**
Age	39.84	**<0.01**
Region*Age	5.15	**<0.01**
Group*Age	10.67	**<0.01**

These variables resulted after eliminating predictor variables having a p-value > 0.01.*Interaction between variables. Bold indicates statistically significant (P-value < 0.01).

(4) QSM ~ region+ age+region* age+group* age+region*group*age+(1|subject)

These findings revealed that there is a significant difference between individuals with 22q11.2 DS and healthy controls when analyzed in each region across age.

Similarly, the backward analysis revealed that, for R2* values, region and age were significant predictors, together with the interactions region-age and group-age (**Table 2**), leading to the model.

(5) $$\begin{array}{c} R2^{*}\sim \;region+age+region*\;age+group*\;age\;+(1\text{|subject}) \end{array}$$

R2* values seem to have a similar behavior compared to QSM in terms of their dependencies.

**Figure 2:**
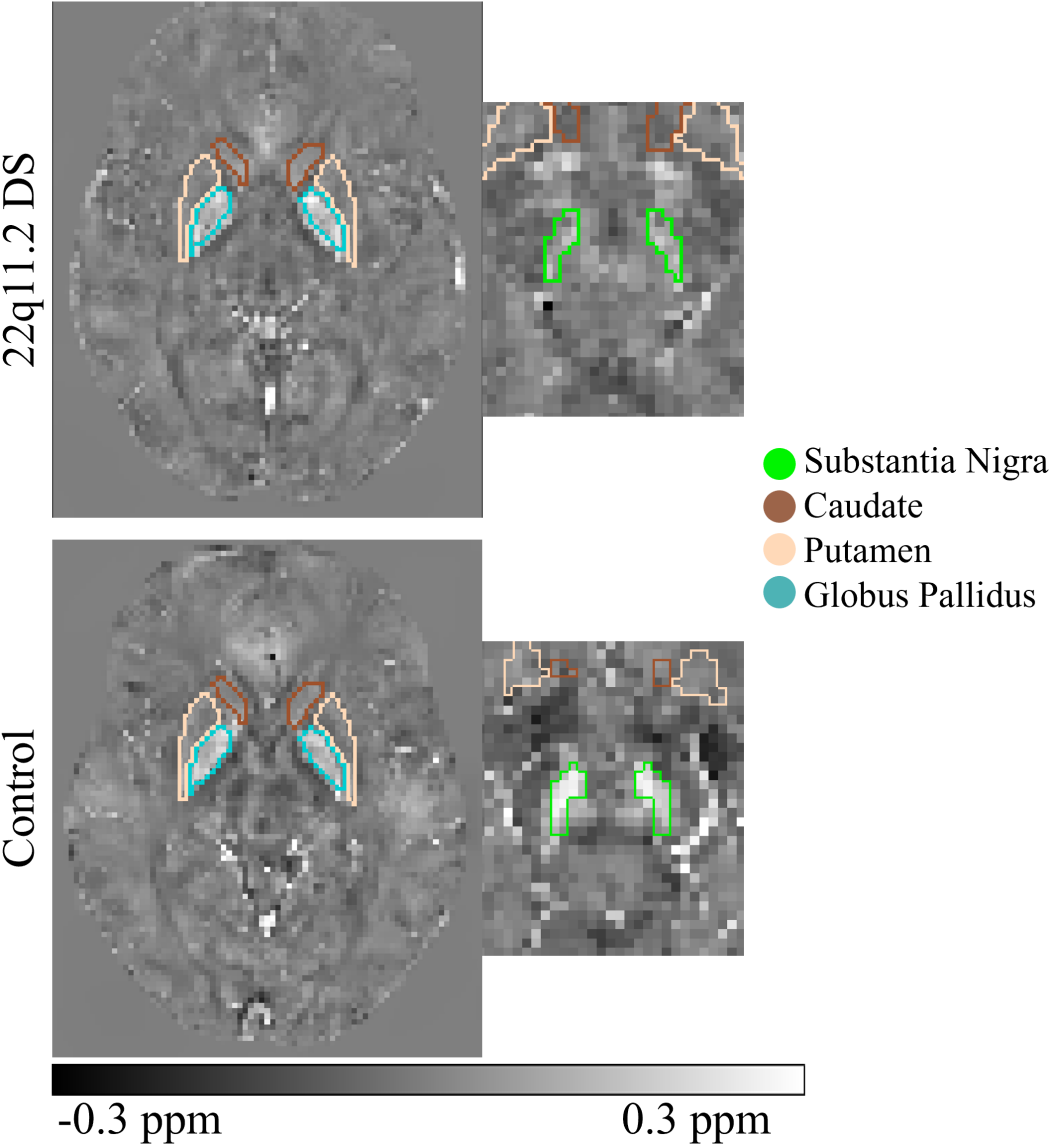
Susceptibility maps of an example of the healthy control group and 22q11.2 DS individuals in substantia nigra, putamen, caudate and globus pallidus.

Model 2: For individuals with 22q11.2 DS, we found that the caudate, putamen and GP (considering SN as the reference region) were significant predictors of the magnetic susceptibility when analyzed across age. This means that the magnetic susceptibility in those regions changes (**Figure 2**) with age, but the rate of change is significantly different when compared between individuals with 22q11.2 DS and healthy controls. This model is robust since the inference is significant in both marginal and random intercept models (**Table 3**).

**Table 3:** Marginal and random intercept model for QSM. For each model, we show the estimated fixed effect variables (considering SN as reference), standard error (std. error) and p-value.

Fixed effect	Random intercept model			Marginal model		
	Estimate	Std. error	P-value	Estimate	Std. error	P-value
(Intercept)	0.028	0.013	0.030	0.028	0.014	0.057
Caudate	-0.020	0.011	0.070	-0.020	0.015	0.190
Putamen	-0.042	0.011	**<0.01**	-0.042	0.015	**<0.01**
GP	0.075	0.011	**<0.01**	0.075	0.019	**<0.01**
Age	0.004	0.001	**<0.01**	0.004	0.001	**<0.01**
Caudate*Age	-0.003	0.0005	**<0.01**	-0.003	0.001	**<0.01**
Putamen*Age	-0.003	0.0005	**<0.01**	-0.003	0.001	**<0.01**
GP*Age	-0.004	0.0005	**<0.01**	-0.004	0.001	**<0.01**
Age*DG	-0.002	0.0003	**<0.01**	-0.002	0.0003	**<0.01**
Caudate*Age*DG	0.002	0.0002	**<0.01**	0.002	0.0003	**<0.01**
Putamen*Age*DG	0.002	0.0002	**<0.01**	0.002	0.0003	**<0.01**
GP*Age*DG	0.002	0.0002	**<0.01**	0.002	0.0004	**<0.01**

DG: Deletion group, GP: Globus Pallidus, *Interaction between the variables. Bold indicates statistically significant (P-value < 0.01).

**Figure 3:**
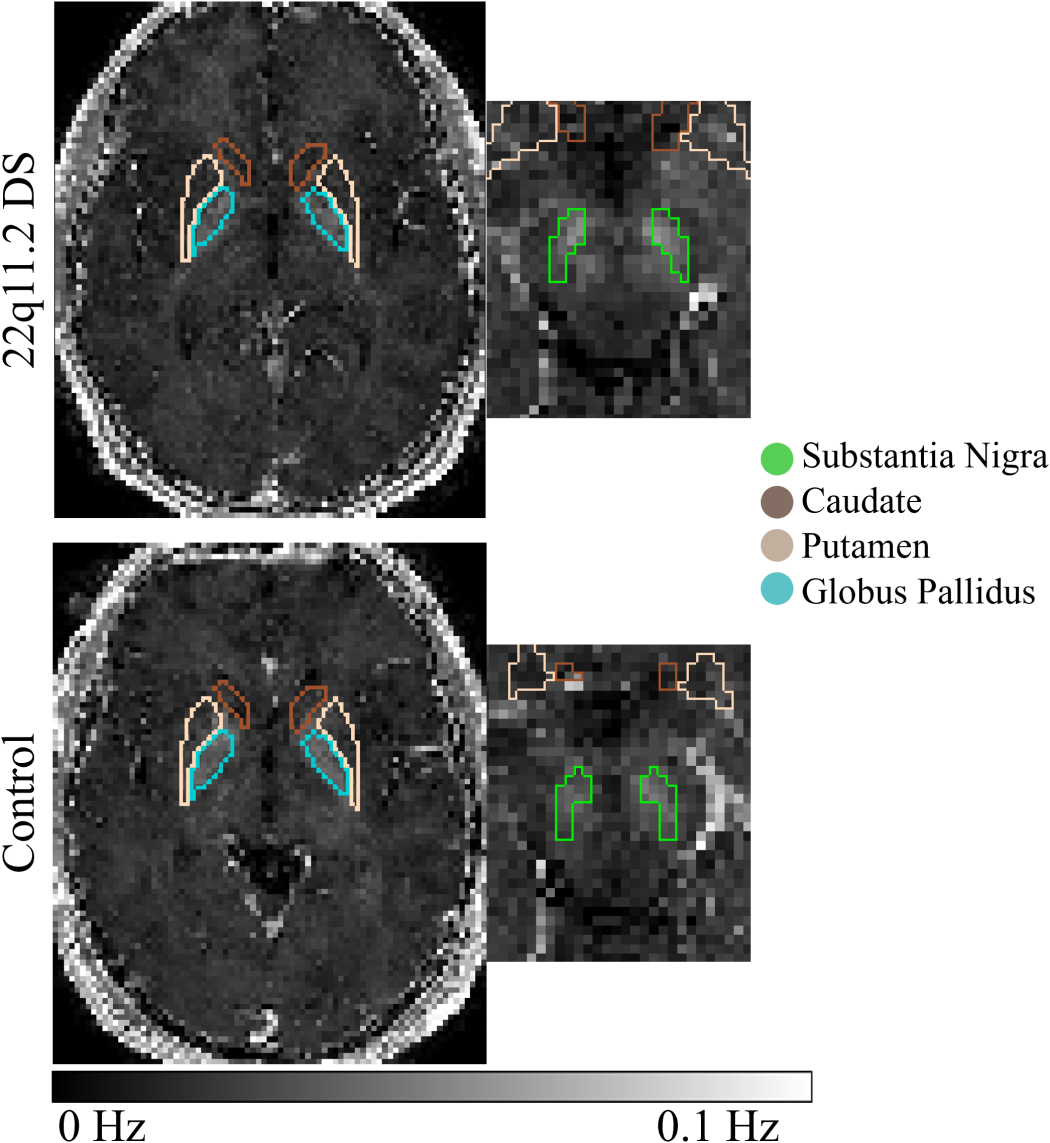
R2* maps of an example of the healthy 22q11.2 DS (first row) and controls (second row) individuals in substantia nigra, putamen, caudate and globus pallidus.

Considering R2* (**Figure 3**), we found that only age was a significant predictor. Additional predictors and interactions showed significance in the random intercept model, but those results could not be confirmed by the marginal model (**Table 4**).

**Table 4:** Marginal and random intercept model for R2*. For each model, we show the estimated fixed effect variables (considering SN as reference), standard error (std. error) and p-value.

Fixed effect	Random intercept model			Marginal model		
	Estimate	Std. error	P-value	Estimate	Std. error	P-value
(Intercept)	0.021	0.001	**<0.01**	0.021	0.002	**<0.01**
Age	0.0004	0.0001	**<0.01**	0.0004	0.0001	**<0.01**
Caudate	-0.004	0.001	**<0.01**	-0.004	0.002	0.078
Putamen	-0.004	0.001	**<0.01**	-0.004	0.002	0.029
GP	0.006	0.001	**<0.01**	0.006	0.003	0.024
Age*Caudate	-0.0002	0.0001	**<0.01**	-0.0002	0.0001	0.013
Age*Putamen	-0.0001	0.0001	**<0.01**	-0.0001	0.0001	0.100
Age*GP	-0.0001	0.0001	**<0.01**	-0.0001	0.0001	0.223
Age*DG	-0.0001	0.00002	**<0.01**	-0.0001	0.00001	**<0.01**

DG: Deletion group, GP: Globus Pallidus. * Interaction between the variables. Bold indicates statistically significant (P-value < 0.01).

Model 3: Considering the models in Equations 4 and 5, we performed a stratified analysis (i.e., for each region) including age and the interaction group-age as cofactors, leading to Equation 6 and Equation 7:

(6) $$\begin{array}{c} QSM_{region}\sim \;\beta_{1}\;age+\beta_{2}\;\;group*age+(1\text{|subject}) \end{array}$$

(7) $$\begin{array}{c} R2_{region}^{*}\sim \;\beta_{1}\;age+\beta_{2}\;\;group*age+(1\text{|subject}) \end{array}$$

**Figure 4:**
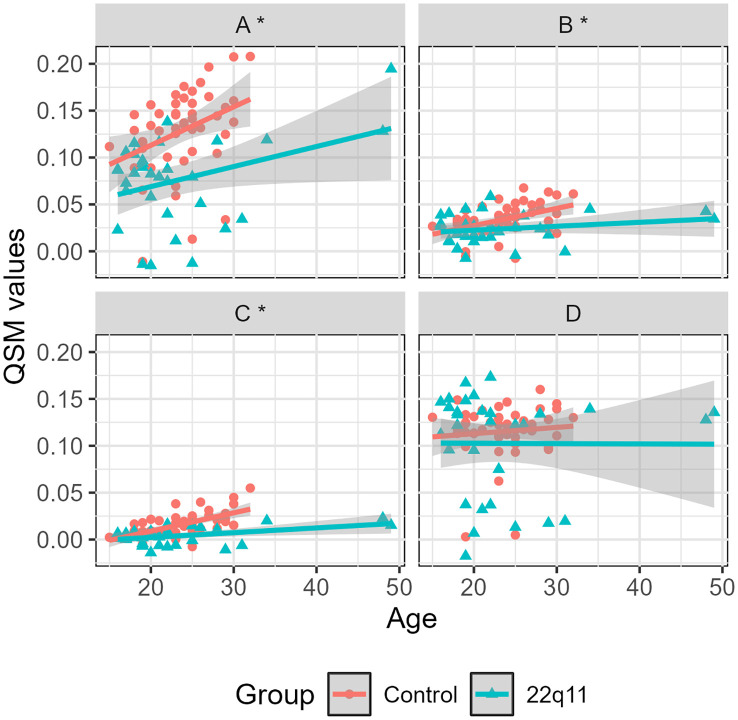
Magnetic susceptibility vs. age for controls and 22q11DS individuals separated by region: substantia nigra **(A)**, caudate **(B)**, putamen **(C)** and globus pallidus **(D)**. Significant differences are marked with an (*).

We found that all individuals presented a significant increase with age in susceptibility values in the SN (β1 = 4.24 x 10-3, p < 0.01), caudate (β1 = 1.13 x 10-3, p < 0.01) and putamen (β1 = 1.28 x 10-3, p < 0.01) (**Table 5**). This effect can be also observed in the linear regressions (**Figure 4**) and the ANCOVA analyses (**Supplementary Tables 1**–**3**). Age showed no statistical significance in the globus pallidus (**Supplementary Table 4**). However, this increase with age is significantly different between groups, being less pronounced for individuals with 22q11.2 DS than healthy controls in the SN (β2 = -2.15 x 10-3, p < 0.01), caudate (β2 = -4.65 x 10-4, p < 0.01) and putamen (β2 = -5.44 x 10-4, p < 0.01) (**Table 5**). This effect can be also observed in the linear regressions (**Figure 4**). The ANCOVA analyses confirmed this finding only for the putamen (Supplementary table 3). We adjusted the p-value using the False Discovery Rate (FDR) correction and we found no changes in the significance of the 4 analyzed regions. Mean and standard values for QSM and R2* values are presented in the **Supplementary Tables 5** and **6**, respectively.

**Table 5:** Estimated *β* values, intercepts, and P-values of a region-specific coefficient regression for all individuals considering age effects based on the random intercept model for QSM described by Equation 6.

Region	Intercept		*β*_1_ (Age)		*β*_2_ (Age*group)	
	Estimate	P-value	Estimate	P-value	Estimate	P-value
Substantia Nigra	0.0275	0.17	4.24x10^-3^	**<0.01**	-2.15x10^-3^	**<0.01**
Caudate	7.64x10^-3^	0.28	1.13x10^-3^	**<0.01**	-4.65x10^-4^	**<0.01**
Putamen	-0.0140	**<0.01**	1.28x10^-3^	**<0.01**	-5.44x10^-4^	**<0.01**
Globus Pallidus	0.1023	**<0.01**	5.54x10^-4^	0.5	-5.46x10^-4^	0.16

Bold indicates statistically significant (P-value < 0.01).

**Figure 5:**
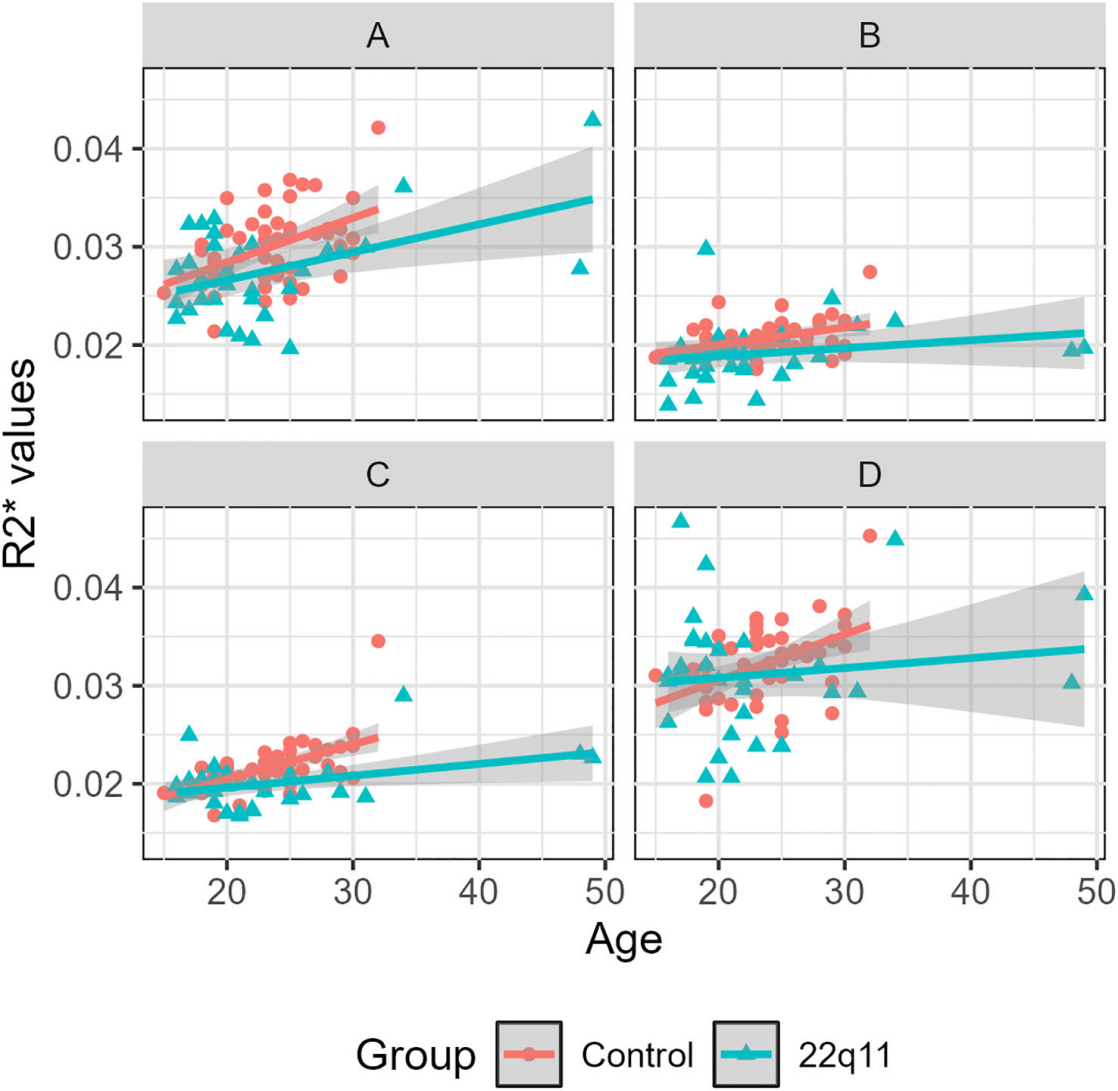
R2* values vs. age for controls and 22q11DS individuals separated by region: substantia nigra **(A)**, caudate **(B)**, putamen **(C)** and globus pallidus **(D)**.

We also found that all individuals presented a significant increase with age in R2* values in the same regions, i.e., SN (*β_1_*= 4.05 x 10^-4^, *p* < 0.01), caudate (*β_1_*= 1.53 x 10^-4^, *p* < 0.01) and putamen (*β_1_*= 2.39 x 10^-4^, *p* < 0.01) (**Table 6**). This effect can be also observed in the linear regressions (**Figure 5**). The ANCOVA analyses confirmed this finding for the SN, putamen and globus pallidus (**Supplementary Tables 7**–**9**). Age showed no statistical significance in the caudate (**Supplementary Table 10**). As observed for magnetic susceptibility, the increase in R2* with age is also different between groups, being again less pronounced for DS individuals than healthy controls in SN (*β_2_* = -1.04 x 10^-4^, *p* < 0.01), caudate (*β_2_* = -6.48 x 10^-5^, *p* < 0.01) and putamen (*β_2_* = -7.83 x 10^-5^, *p* < 0.01) (**Table 6**). This effect can be also observed in the linear regressions (**Figure 5**). The ANCOVA analyses confirmed this finding only for the putamen (**Supplementary Table 8**). We also adjusted the p-value using the FDR correction and found no changes in the significance for the 4 regions analyzed.

**Table 6:** Estimated *β* values, intercepts, and P-values of a region-specific coefficient regression for all individuals considering age effects based on the random intercept model for R2* described by Equation 7.

Region	Intercept		*β*_1_ (Age)		*β*_2_ (Age*group)	
	Estimate	P-value	Estimate	P-value	Estimate	P-value
Substantia Nigra	0.0205	**<0.01**	4.05x10^-4^	**<0.01**	-1.04x10^-4^	**<0.01**
Caudate	1.70x10^-2^	**<0.01**	1.53x10^-4^	**<0.01**	-6.48x10^-5^	**<0.01**
Putamen	1.61x10^-2^	**<0.01**	2.39x10^-4^	**<0.01**	-7.83x10^-5^	**<0.01**
Globus Pallidus	2.67x10^-2^	**<0.01**	2.39x10^-4^	0.018	-5.88x10^-5^	0.21

Bold indicates statistically significant (P-value < 0.01).

The Mann Whitney U test showed no significant difference between the volumes of each group (**Supplementary Table 11**).

## 4 Discussion

Using a novel approach such as QSM, we quantified the magnetic susceptibility of individuals with 22q11 deletion syndrome and compared it with that obtained from healthy controls in dopaminergic regions. For all individuals, we found a positive correlation between age and both QSM and R2* values in nigrostriatal regions (**Tables 5**, **6**). Similar results have been reported in previous studies (60–62). However, we found that these variations differ between groups for both QSM and R2*. Indeed, individuals with 22q11.2 DS present lower rates of change compared to controls (**Figures 4**, **5**) in the SN, caudate and putamen. This observation is quantitatively supported by the negative β_2_ values in **Tables 5**, **6**. On average, individuals with 22q11.2 DS showed lower susceptibility and R2* values than controls in the SN, caudate and putamen (**Figure 1**), particularly for individuals over approximately 20 years old (**Figures 4**, **5**). A reduction in both, magnetic susceptibility and R2* values, suggests a decrease in iron concentrations (29). Consequently, over approximately 20 years old, individuals with 22q11.2 deletion syndrome appear to have reduced iron concentrations compared to healthy controls in the SN, caudate and putamen. 

The relation between dopamine and iron is still not entirely understood, particularly in pathologies related with dopamine dysfunctions (63–65). One limitation to such understanding was the absence of a tool that could quantify *in vivo* the subtle changes in iron concentrations that apparently occur in small brain nuclei in dopamine-related diseases. The development of the QSM technique has opened a window to study *in vivo* iron distributions, particularly in pathological contexts, such as diseases related to dopamine dysfunctions. In Parkinson’s disease, Pyatigorskaya et al. has reported increased susceptibility in striatal regions (20), while Butcher et al., found a reduction in dopaminergic striatal activity, especially in the putamen (11). In first episode psychosis, a reduction in magnetic susceptibility was found in the globulus pallidum externa (47). Similarly, Vano et al. (21) showed an association between lower levels of susceptibility values (and therefore lower iron concentrations) in the SN and ventral tegmental area and striatal hyperdopaminergia in schizophrenia (22). On the other hand, Ravanfar et al. (66) reported elevated iron levels in the putamen of individuals with chronic schizophrenia. Kegeles et al. suggests an elevated dopamine disfunction of striatal regions in schizophrenia individuals (67). Further research is needed to better understand the relationship between iron accumulation and dopamine levels.

22q11.2 DS confers a high risk to develop neuro-psychiatric disorders such as schizophrenia or Parkinson’s during different periods in their lives. This corresponds to opposite dysfunctions in the dopaminergic system, particularly a hyperdopaminergic state in psychosis in young adulthood, followed by a hypodopaminergic state in Parkinson’s later in life (4, 8, 11). This behavior is also observed by analyzing neuromelanin, a byproduct of the dopamine synthesis and an important contributor to maintain the iron homeostasis in the brain. Researchers using the neuromelanin-based contrasts technique in Parkinson’s disease have shown decreases in neuromelanin content (14), unlike the increase found in psychosis (68).

The significant decrease of magnetic susceptibility that we found in dopamine-rich regions in 22q11.2 DS individuals (i.e., SN, caudate and putamen) follows a similar path of previous studies found in psychosis (47) and the opposite direction of that found in Parkinson’s disease (34). This behavior might be related to a disfunction in dopaminergic pathways in 22q11.2 DS individuals. A recent PET study in 22q11.2 DS using a pre-synaptic marker of dopamine concentration (12) showed that the neurochemical characterization of striatal regions in 22q11.2 DS is consistent with alterations typically found in psychosis (12). Another PET study (11) with a dopamine transporter radioligand ^11^C-DTBZ used for the assessment of Parkinson’s found that 22q11.2 DS individuals presented a reduction in the striatal signal as expected in individuals with Parkinson’s disease (69). However, 22q11.2 DS individuals at risk of Parkinson’s disease presented a mean elevated striatal of the radioligand relative to controls (11). These dopamine changes reported in the 22q11.2 DS literature associated to psychosis and Parkinson’s disease might be the result of a temporal evolution process. Indeed, the PET study by Rogdaki et al. was performed in a cohort of 22q11.2 DS individuals with psychosis and a mean age of 26.1 years (12), whereas the PET study by Butcher et al. was performed in a cohort of 22q11.2 DS individuals with Parkinson’s and a mean age of 41.5 years (11). Further studies must be done to link the relation between magnetic susceptibility and dopamine disfunction across the age.

Our results show that brain iron deposition tends to increase with age across all participants, consistent with previous findings (70). If individuals with 22q11.2 DS follow a trajectory similar to that independently reported in individuals with psychosis and Parkinson’s disease, they would be expected to show reduced brain iron concentrations relative to healthy controls during early adulthood—potentially contributing to their increased risk of psychosis at this stage—followed by a subsequent accumulation of iron, which may underlie their elevated risk for Parkinson’s disease later in life. Our experiments captured aspects of this behavior. Individuals with 22q11.2 DS exhibited lower magnetic susceptibility and R2* values, indicating reduced iron concentrations. However, they showed a slightly attenuated increase in iron accumulation with age compared to controls, as reflected by the negative β_2_ values obtained for QSM and R2* (**Tables 5**, **6**). This pattern contrasts with that typically observed in Parkinson’s disease, an increased iron accumulation in dopaminergic regions (34). The temporal trajectory of iron changes, however, remains unclear. Xuan et al. reported that individuals with middle- to late-onset Parkinson’s disease exhibit increased iron content in the putamen, whereas those with early-onset Parkinson’s disease do not (71). Our sample size did not allow for a reliable examination of susceptibility changes beyond 30 years of age. Future longitudinal studies will be necessary to confirm our proposed hypothesis regarding the temporal evolution of iron accumulation.

Unlike what was found in (12), we did not find any volumetric changes in the analyzed nuclei (**Supplementary Table 11**).

Our study has some limitations. It has a relatively low number of participants, allowing us to perform region of interest analyses based on the literature rather than a whole brain approach. Around 15% of individuals initially recruited had to be excluded from the study due to motion-related artifact. Future research works might develop mitigation strategies to control motion in QSM studies.

Since our results showed a decrease in iron concentration at nigrostriatal regions, we hypothesize that a dopaminergic disfunction might be observed in 22q11.2DS individuals.

## 5 Conclusions

All individuals showed age-related increases in susceptibility and R2* values in the dopaminergic nuclei (caudate, putamen, and SN). However, the rate of increase was significantly lower in individuals with 22q11.2 deletion syndrome compared to healthy controls. This suggests that, over time, 22q11.2 DS individuals accumulate less iron in these nuclei than healthy controls. Different time trajectories of iron in these nuclei suggest that individuals with 22q11.2 DS may experience changes in dopaminergic status.

## Data Availability

The datasets presented in this article are not readily available due to medical confidentiality. Requests to access the datasets should be directed to ctejos@uc.cl.
