# Supplementary material for: Quantitative susceptibility mapping of deep brain nuclei in 22q11.2 deletion syndrome
Source: Front Psychiatry. 2026 Jan 9;16:1652700. doi: 10.3389/fpsyt.2025.1652700 (PMC12829479; doi:10.3389/fpsyt.2025.1652700)
Supplement: Supplementary file 1 [file SupplementaryFile1.docx]

Supplementary Material


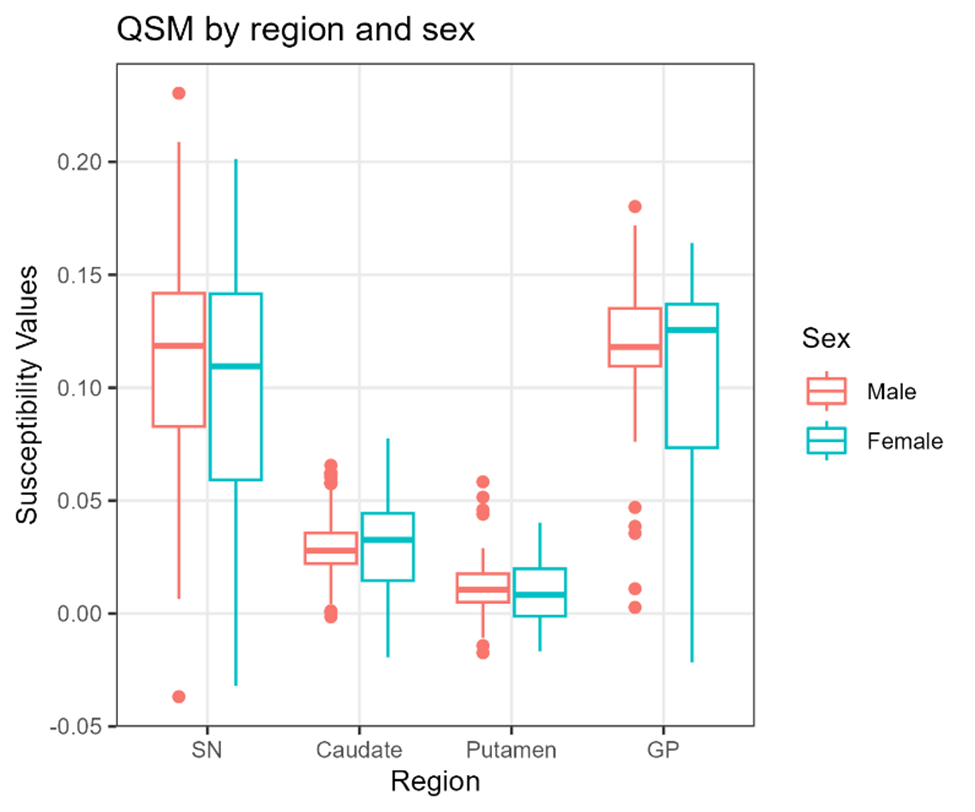


**Supplementary Figure 1.** Boxplots of magnetic susceptibility by sex for each region.

**Supplementary table 1.** Coefficients and p-values of variables of interest from the ANCOVA analysis of magnetic susceptibility (QSM) in the substantia nigra.

| **Variable** | **Coefficient (ppm)** | **P-values** |
| --- | --- | --- |
| **Age** | 2.79x10^-3^ | **<0.01** |
| **Group** | -4.67x10^-2^ | **<0.01** |
| **Sex** | -1.26x10^-2^ | 0.12 |
| **Volume** | -9.48x10^-5^ | 0.38 |

**Supplementary table 2.** Coefficients and p-values of variables of interest from the ANCOVA analysis of magnetic susceptibility (QSM) in the caudate.

| **Variable** | **Coefficient (ppm)** | **P-value** |
| --- | --- | --- |
| **Age** | 1.73x10^-3^ | **<0.01** |
| **Group** | -5.54x10^-2^ | 0.05 |
| **Sex** | 2.66x10^-3^ | 0.32 |
| **Volume** | -3.12x10^-5^ | **<0.01** |
| **Age * Group** | -1.60x10^-3^ | 0.02 |
| **Volume * Group** | 4.57x10^-5^ | **<0.01** |

**Supplementary table 3.** Coefficients and p-values of variables of interest from the ANCOVA analysis of magnetic susceptibility (QSM) in the putamen.

| **Variable** | **Coefficient (ppm)** | **P-value** |
| --- | --- | --- |
| **Age** | 1.97x10^-3^ | **<0.01** |
| **Group** | 3.39x10^-2^ | 0.33 |
| **Sex** | 4.40x10^-4^ | 0.80 |
| **Volume** | -1.28x10^-4^ | 0.02 |
| **Age * Group** | -1.56x10^-3^ | **<0.01** |
| **Volume * Group** | 2.07x10^-5^ | **<0.01** |

**Supplementary table 4.** Coefficients and p-values of variables of interest from the ANCOVA analysis of magnetic susceptibility (QSM) in the globus pallidus.

| **Variable** | **Coefficient (ppm)** | **P-value** |
| --- | --- | --- |
| **Age** | 1.22x10^-4^ | 0.96 |
| **Group** | -0.27 | **<0.01** |
| **Sex** | 1.41x10^-3^ | 0.96 |
| **Volume** | -1.82x10^-4^ | **<0.01** |
| **Age * Group** | 8.89 x10^-6^ | 0.99 |
| **Volume * Group** | 3.73x10^-4^ | **<0.01** |

**Supplementary table 5.** Mean and standard values of QSM values (ppm) for each region, separated by groups.

|  | **Control** | | **22q11** | |
| --- | --- | --- | --- | --- |
| **Region** | **Mean** | **Standard deviation** | **Mean** | **Standard deviation** |
| **SN** | 0.128 | 0.046 | 0.075 | 0.049 |
| **Caudate** | 0.034 | 0.017 | 0.023 | 0.017 |
| **Putamen** | 0.016 | 0.013 | 0.003 | 0.009 |
| **GP** | 0.115 | 0.029 | 0.101 | 0.055 |

**Supplementary table 6.** Mean and standard values of the R2* values (Hz) for each region, separated by groups.

|  | **Control** | | **22q11** | |
| --- | --- | --- | --- | --- |
| **Region** | **Mean** | **Standard deviation** | **Mean** | **Standard deviation** |
| **SN** | 0.030 | 4.22x10^-3^ | 0.027 | 5.38x10^-3^ |
| **Caudate** | 0.021 | 1.95x10^-3^ | 0.019 | 3.33x10^-3^ |
| **Putamen** | 0.022 | 2.57x10^-3^ | 0.020 | 2.68x10^-3^ |
| **GP** | 0.032 | 4.17x10^-3^ | 0.031 | 6.63x10^-3^ |

**Supplementary table 7.** Coefficients and p-values of variables of interest from the ANCOVA analysis of R2* in the substantia nigra.

| **Variable** | **Coefficient (Hz)** | **P-values** |
| --- | --- | --- |
| **Age** | 3.36x10^-4^ | **<0.01** |
| **Group** | -1.74x10^-3^ | **<0.01** |
| **Sex** | -1.69x10^-3^ | 0.02 |
| **Volume** | -2.49x10^-5^ | 0.02 |

**Supplementary table 8.** Coefficients and p-values of variables of interest from the ANCOVA analysis of R2* in the putamen.

| **Variable** | **Coefficient (Hz)** | **P-value** |
| --- | --- | --- |
| **Age** | 3.61x10^-4^ | **<0.01** |
| **Group** | -4.60x10^-4^ | 0.89 |
| **Sex** | -2.33x10^-5^ | 0.95 |
| **Volume** | -3.42x10^-6^ | **<0.01** |
| **Age * Group** | -2.51x10^-4^ | **<0.01** |
| **Group * Volume** | 2.33 x10^-6^ | 0.10 |

**Supplementary table 9.** Coefficients and p-values of variables of interest from the ANCOVA analysis of R2* in the globus pallidus.

| **Variable** | **Coefficient (Hz)** | **P-value** |
| --- | --- | --- |
| **Age** | 2.18x10^-4^ | **<0.01** |
| **Group** | -3.16x10^-4^ | 0.70 |
| **Sex** | -2.49x10^-3^ | **<0.01** |
| **Volume** | -9.62x10^-6^ | 0.05 |

**Supplementary table 10.** Coefficients and p-values of variables of interest from the ANCOVA analysis of R2* in the caudate.

| **Variable** | **Coefficient (Hz)** | **P-values** |
| --- | --- | --- |
| **Age** | 9.76x10^-5^ | 0.011 |
| **Group** | -1.59x10^-3^ | **<0.01** |
| **Sex** | 4.06x10^-4^ | 0.43 |
| **Volume** | -2.83x10^-7^ | 0.76 |

**Supplementary table 11.** Results of the Mann–Whitney test comparing regional volumes between healthy controls and individuals with 22q11.2 DS

| **Region** | **P-value** |
| --- | --- |
| Substantia Nigra | 0.41 |
| Caudate | 0.08 |
| Putamen | 0.10 |
| Globus Pallidus | 0.06 |
